# Mitochondrial Uncoupling Inhibits p53 Mitochondrial Translocation in TPA-Challenged Skin Epidermal JB6 Cells

**DOI:** 10.1371/journal.pone.0013459

**Published:** 2010-10-18

**Authors:** Fei Wang, Xueqi Fu, Xia Chen, Xinbin Chen, Yunfeng Zhao

**Affiliations:** 1 Department of Pharmacology, Toxicology and Neuroscience, LSU Health Sciences Center in Shreveport, Shreveport, Louisiana, United States of America; 2 College of Life Science, Jilin University, Changchun, People's Republic of China; 3 Department of Surgical and Radiological Sciences, University of California Davis, Davis, California, United States of America; Yale Medical School, United States of America

## Abstract

The tumor suppressor p53 is known to be able to trigger apoptosis in response to DNA damage, oncogene activation, and certain chemotherapeutic drugs. In addition to its transcriptional activation, a fraction of p53 translocates to mitochondria at the very early stage of apoptosis, which eventually contributes to the loss of mitochondrial membrane potential, generation of reactive oxygen species (ROS), cytochrome *c* release, and caspase activation. However, the mitochondrial events that affect p53 translocation are still unclear. Since mitochondrial uncoupling has been suggested to contribute to cancer development, herein, we studied whether p53 mitochondrial translocation and subsequent apoptosis were affected by mitochondrial uncoupling using chemical protonophores, and further verified the results using a siRNA approach in murine skin epidermal JB6 cells. Our results showed that mitochondrial uncoupling blocked p53 mitochondrial translocation induced by 12-*O*-tetradecanoylphorbol 13-acetate (TPA), a known tumor promoter to induce p53-mediated apoptosis in skin carcinogenesis. This blocking effect, in turn, led to preservation of mitochondrial functions, and eventually suppression of caspase activity and apoptosis. Moreover, uncoupling protein 2 (UCP2), a potential suppressor of ROS in mitochondria, is important for TPA-induced cell transformation in JB6 cells. UCP2 knock down cells showed enhanced p53 mitochondrial translocation, and were less prone to form colonies in soft agar after TPA treatment. Altogether, our data suggest that mitochondrial uncoupling may serve as an important regulator of p53 mitochondrial translocation and p53-mediated apoptosis during early tumor promotion. Therefore, targeting mitochondrial uncoupling may be considered as a novel treatment strategy for cancer.

## Introduction

Most of the cell's redox reactions take place in mitochondria, which supply cellular energy by oxidating the major products of glucose, pyruvate, and NADH. Substrate oxidation in this cellular respiration process generates a proton gradient across the mitochondrial inner membrane that establishes the electrochemical potential (Δ*ψ*_m_). The energy contained in Δ*ψ*_m_ is mainly used for ATP synthesis (oxidative phosphorylation). However, not all of the energy available in the electrochemical gradient is coupled to ATP synthesis. Some of the energy is consumed by “proton leak” reactions, by which protons pumped out of the matrix are able to reflow back along the proton gradient through proton conductance pathways in the inner membrane that bypass the ATP synthase. As a result, the energy derived from the metabolic oxidation reaction is dissipated as heat [1]–[4]. The nonproductive proton leak termed mitochondrial uncoupling is physiologically important and accounts for 20–25% of basal metabolic rate [5], [6].

Notably, the impact of mitochondrial uncoupling on cellular physiology is not restricted to normal cells. Mitochondrial uncoupling also plays an important role in the reprogramming of cancer cell metabolism [7]. ROS generation is involved in the regulation of many physiological processes in cancer cells which promotes further genomic instability; and upregulates signaling pathways of cellular growth and proliferation. However, if produced excessively, ROS may also be harmful to the cell by initiating cell death pathways [8], [9]. Hence, a well controlled ROS level in cancer cells is of critical importance for tumor cell physiology, growth and survival [10]. Since mitochondrial uncoupling has been suggested to have a natural antioxidant effect that increases respiratory rates and thus attenuates ROS generation [11]–[13], it is not surprising that high levels of mitochondrial uncoupling are found in various chemoresistant cancer cell lines, which may provide a prosurvival advantage to tumor cells [14], [15].

p53, considered as a pivotal tumor suppressor, can initiate apoptosis in response to cellular stress stimuli (e.g. drugs, irradiation, UV, hypoxia) and the expression of viral or cellular oncogenes [16]. In addition, mutations of p53 are found in more than 50% of all human cancers [17]. Interestingly, recent studies have suggested that p53 and mitochondrial uncoupling are contradictory during apoptosis. It was reported that the Warburg effect in leukemia cells is mediated by mitochondrial uncoupling associated with UCP2 activation, and dissipation of Δ*ψ*_m_ by protonophore carbonyl cyanide 3-chlorophenylhydrazone (CCCP) opposes the onset of apoptosis [18]. Furthermore, overexpression of UCP2 in several colon cancer cell lines is able to promote chemoresistance which is, at least in part, resulting from the inhibition of p53-induced apoptosis by posttranslational modification of p53 [19]. These data suggests a strong relationship between mitochondrial uncoupling and the p53-mediated apoptotic pathway in the tumor metabolism network.

Our previous studies using the skin carcinogenesis model revealed that during early tumor promotion, the tumor suppressor p53 was activated after tumor promoter treatment, and a fraction of p53 translocated into mitochondria which preceded its nuclear translocation. Moreover, the mitochondrial p53 targeted the primary antioxidant defense enzyme, manganese superoxide dismutase (MnSOD), leading to suppression of its superoxide scavenging activity, as well as, increases in ROS levels [20]–[23]. Thus, in addition to the direct apoptotic activity of mitochondrial p53, the ability to induce ROS accumulation might serve as a positive feed-back loop and play an essential role in the p53-mediated apoptosis pathway [24]. However, as a major contributor to cancer survival, whether mitochondrial uncoupling could exert an anti-apoptotic influence on TPA-induced skin tumor promotion, and the precise mechanisms by which mitochondrial uncoupling may interact with p53 mitochondrial translocation and associated cell apoptosis pathways are not known and have been explored here.

## Materials and Methods

### Cell line, reagents, and treatment

Murine epidermal JB6 P+ (CL 41, promotable by TPA treatment) cells were established and maintained as previously described [25]. p53 is wild-type in this cell line [26]. The cells were grown in Essential Modified Eagle's Medium (EMEM) supplemented with 4% fetal bovine serum, 2 mM of L-glutamine, 50 µg/ml penicillin and 50 µg/ml streptomycin. Murine skin keratinocyte 308 cells were growth in S-MEM medium supplemented with 8% Chelexed FBS, 2 mM of L-glutamine, 0.1 mM nonessential amino acids, 50 µM Ca^2+^, and 50 µg/ml penicillin and 50 µg/ml streptomycin. 308 cells carry mutated H-ras at codon 61 but wild-type in p53 [23]. 20 mM carbonyl cyanide 4-(trifluoromethoxy)phenylhydrazone (FCCP) and carbonyl cyanide 3-chlorophenylhydrazone (CCCP) (both were purchased from Sigma, St. Louis, MO) stock solutions were prepared in dimethylsulfoxide (DMSO). 12-*O*-tetradecanoylphorbol 13-acetate (TPA, purchased from Sigma) was prepared as 1 mM stock solution in DMSO. All of the stock solutions were diluted directly in the cell culture medium, and the final concentrations of FCCP, CCCP, and TPA used were 10 µM, 5 µM, and 100 ng/ml, respectively. For all of the pretreatment studies, the reagents were removed before TPA treatment.

### Cell transfection and gene knockdown

A combined pool of 3 target-specific siRNAs and specific siRNA transfection reagent (Santa Cruz) were used to inhibit UCP2 expression. For p53 knock down experiments, pBabe-U6 plasmids encoding shRNA that specifically inhibit p53 expression [27] were used together with FuGENE HD transfection reagent (Roche). Healthy and subconfluent JB6 P+ cells were transfected for 48 h before further analysis. A fluorescein conjugated non-targeting siRNA (Santa Cruz) and a GFP-encoding control plasmid was used to monitor the transfection efficiency. The expression of target genes was further examined by Western blot analysis with specific antibodies.

### Detection of mitochondrial membrane potential

Five thousand JB6 cells were seeded in 96-well plates with 150-µl medium. Twenty-four hours after plating, cells were treated as indicated. After washing with PBS, cells were incubated in fresh medium containing 2 µg/ml of 5,5′,6,6′-tetrachloro-1,1′,3,3′-tetraethylbenzimidazol-carbocyanine iodide (JC-1, Molecular Probes, Eugene, Oregon) for 30 min. The dye was then removed; and cells were washed with PBS. Fluorescence intensity was measured immediately using fluorescence spectrometry (Synergy HT, BioTek, Winooski, VT). For JC-1 green, Ex = 485, Em = 528; for JC-1 red, Ex = 530, Em = 590. The fluorescence signals from the cells only (no JC-1 dye added) were used to subtract the sample values from each corresponding well. The ratio of the red to green fluorescence of JC-1 was calculated. Experiments were repeated for three times and at the least triplicate samples were included in each experiment.

### Detection of mitochondrial ROS generation

The mitochondrial levels of ROS were assayed using the mitoSOX Red dye. Briefly, five thousand JB6 P+ cells were seeded in a 96-well plate and incubated overnight. Twenty-four hours after plating, cells were treated as indicated in each experiment. After washing with warm PBS, cells were incubated with fresh medium containing 5 µM mitoSOX Red (Molecular Probes, Eugene, OR) for 15 min at 37°C. The fluorescence intensity was measured at excitation/emission of 530/590 nm using fluorescence spectrometry (Synergy HT,). The cells only sample (no mitoSOX dye added) was used as the background. Experiments were repeated for three times and at least triplicate samples were included in each experiment.

### Immunofluorescent staining of p53

Ten thousand JB6 or 308 cells were seeded in eight-well Lab-Tek chamber slides w/cover (Nalge Nunc International, Naperville, IL) in 400 µl medium per well and incubated overnight. Twenty-four hours after plating, cells were incubated with 200 nM of MitoTracker Red CMX-Ros (Molecular Probes) in culture medium for 30 min. Afterwards the dye was removed, and cells were treated as indicated. The cells were washed and fixed in 4% formaldehyde solution for 15 min at room temperature. After rinsing with cold PBS, cells were permeabilized with 0.5% Triton X-100 for 10 min at room temperature. After blocking, an anti-p53 antibody (Ab-11, Calbiochem) was added (1∶64 dilution) and incubated at 37°C for 1 hour followed by incubation with an anti-mouse IgG-FITC (Sigma, 1∶128 dilution) for 1 hour. After removal of antibodies, the cells were rinsed with PBS and mounted with 90% glycerol. Fluorescence was immediately observed using a wide-field inverted microscope (Nikon Eclipse TE300).

### Isolation of mitochondrial fraction from JB6 and 308 cells

After treatment, cells were suspended in 2 ml mitochondria isolation buffer [0.225 M mannitol, 0.075 M sucrose, 1 mM EGTA (pH adjusted to 7.4 with 0.5 M Tris)] in a 10-ml Wheaton homogenizer tube and carefully homogenized for 30 strokes on ice. The cell debris was removed by centrifugation at 2,500 rpm (∼600×g) twice for 5 min. The supernatant was filtered through a nylon screen cloth (Small Parts, Inc., Miami Lakes, FL) and then centrifuged at 10,000 rpm (∼9,000×g) for 10 min. The supernatant was kept as the *cytosolic fraction* and the pellet was washed by adding 0.5 ml of mitochondria isolation buffer and centrifuged at 10,000 rpm for 5 min. This washing step was repeated twice. The mitochondrial pellet was resuspended in 50–100 µl of mitochondria isolation buffer containing protease inhibitor cocktail (Research Products International Corp. Mount Prospect, IL). The purity of the mitochondrial and cytosolic fractions was further examined by Western blot analysis.

### Mitochondrial Complex I activity assay

Complex I specific activities were measured as described by Birch-Machin et al. [28] with slight modifications [29]. Mitochondrial samples isolated from JB6 cells were subjected to three fast freeze-thaw cycles in hypotonic buffer. The protein concentration was measured and adjusted to 1.33 µg/µl before the assay. The assay mixtures, which contained 25 mM potassium phosphate buffer (pH 7.2), 5 mM MgCl_2_, 2 mM KCN, 2.5 mg/ml bovine serum albumin (fraction V), 0.13 mM NADH, 65 mM coenzyme Q1, and 2 mg/ml antimycin A, were incubated at 30°C for 1 min. Mitochondria were added to initiate the reaction, and the initial rate of NADH oxidation was monitored at 340 nm for 1 min (ΔA). The reaction was inhibited by 2 µl of 2 mg/ml rotenone and the rate of NADH oxidation was monitored for 1 min (ΔA_r_). The relative complex activity was calculated according to the following formula: ΔA-ΔA_r_.

### Western blot analysis

Total cell lysate was prepared by sonicating cells in RIPA buffer supplemented with protease inhibitor cocktail for 10 s. Mitochondrial fractions were prepared as described above and 0.5% Triton X-100 was added to the final solutions. Protein samples were separated on a 10% or 15% SDS-PAGE gel. The following antibodies were used: anti-p53 (Ab11, Calbiochem), anti-MDM2 (Ab2, Calbiochem), anti-Bax (P19, Santa Cruz), anti-cytochrome *c* (A8, Santa Cruz), and anti-active caspase-3 (AB3623, Chemicon). For loading controls, antibodies against Glyceraldehyde 3-phosphate dehydrogenase (GAPDH) (6C5) and succinate dehydrogenase complex subunit B (SDHB) (FL280) were used (both purchased from Santa Cruz).

### Caspase 3 Activity assay

Total cell lysate was prepared and caspase 3 activities were detected using CasPASE apoptosis assay kit (G-Biosciences, St Louis, MO) following the manufacturer's instructions.

### Nucleosome fragmentation assay

Cellular fragmentation was performed following the manufacturer's instructions. DNA fragmentation in cell lysate was detected using Cell Death Detection ELISA (Roche, Indianapolis, IN). This method applies an anti-histone antibody to capture the histone-DNA fragments, then applies a HRP-labeled anti-DNA antibody to detect fragmented DNA.

### Soft agar assay

Soft agar assay was performed as described by Colburn et al. [30] with slight modifications. 0.5% Agar mix (40 ml melted 1.25% agar solution, 40 ml 2×EMEM, 10 ml FBS, 10 ml PBS, 1 ml glutamine, 50 µl penicillin & streptomycin) was prepared and kept in a 50°C water bath. Bottom agar was prepared by adding desired treatments (e.g. 6.66 ng/ml TPA) to the 0.5% agar mix. Top agar was prepared by diluting 1 fraction of 1×10^5^ cells/ml single cell suspension with 2 fractions of 0.5% agar mix and desired treatments. 2.5 ml of bottom agar and 0.75 ml of top agar was laid into each well of the 6-well plates. Cells were incubated in a humidified 37°C, 5% CO_2_ incubator for 2 weeks. Cells were then stained with 0.25 mg/ml neutral red overnight, and the number of colonies were counted and plotted.

### Statistical analysis

Statistical analysis was performed using one-way ANOVA followed by Newman-keuls post-test. Data are reported as means ± standard error (S.E.M.). p<0.05 was considered significant.

## Results

### TPA induced p53 mitochondrial translocation, mitochondrial dysfunction and apoptosis in JB6 P+ cells

**Figure 1:**
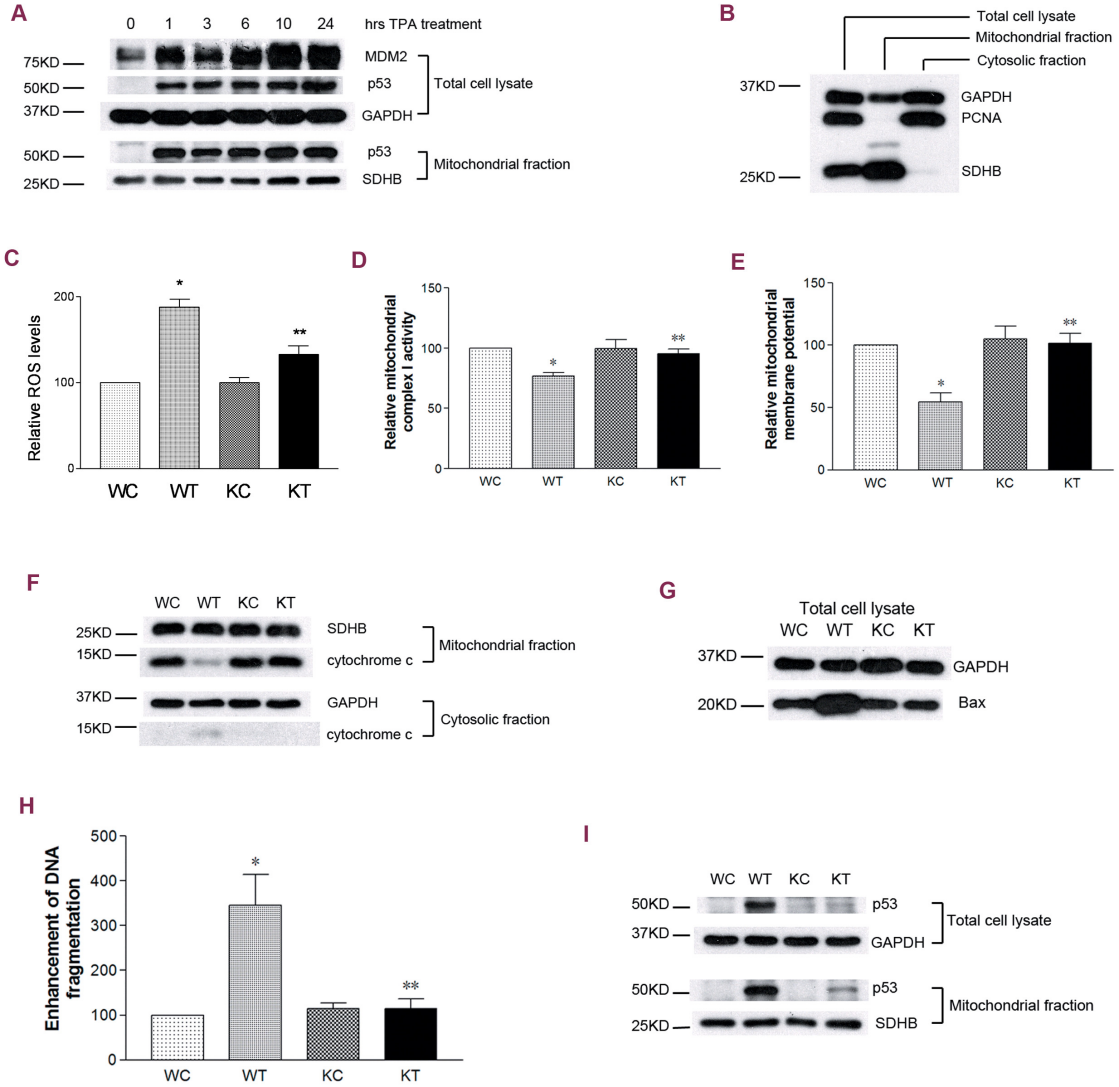
TPA induced p53 mitochondrial translocation, mitochondrial dysfunction and apoptosis in JB6 P+ cells. WC: wild-type JB6 P+ cells treated with DMSO (control); WT: wild-type JB6 P+ cells treated with 100 nM TPA; KC: p53-knocdown JB6 P+ cells treated with DMSO (control); KT: p53-knockdown JB6 P+ cells treated with 100 nM TPA; GAPDH or SDHB served as the loading control. (A) A time course study of TPA-induced cellular and mitochondrial p53 accumulation, as well as, downstream MDM2 expression. (B) Qualification of mitochondrial fractions. (C) p53 knockdown and ROS levels. Cells were treated for 1 h. (D) p53 knockdown and mitochondrial complex I activities. Cells were treated for 1 h. (E) p53 knockdown and mitochondrial membrane potential. Cells were treated for 1 h. (F) p53 knockdown and cytochrome *c* release. Cells were treated for 6 h. (G) p53 knockdown and cellular Bax levels. Cells were treated for 6 h. (H) p53 knockdown and cell death levels revealed by DNA fragmentation assays. Cells were treated for 6 h. (I) p53 mitochondrial translocation verified by gene knockdown assays. *: significant difference compared to WC; **: significant difference compared to WT.

Initially, a time course study was performed to detect p53 accumulation in the whole cell lysate. The increase in p53 protein levels was observed as early as 1 h after 100 nM TPA treatment and remained at high levels for the duration of the 24 h treatment (Fig. 1A). As a major p53 transcriptional target protein, MDM2 showed a rapid response with a significant increase at 1 h, a slight decline at 3 h, and increased again at later time points after the TPA treatment (Fig. 1A). Our previous studies have revealed that a fraction of p53 translocated to mitochondria during TPA-induced tumor promotion [22]. Herein, we performed a detailed time course study. After treatment, cells were subjected to a fragmentation procedure by which mitochondrial and cytosolic fractions were collected and qualified with specific protein markers. In the mitochondrial fractions, PCNA, a nuclear marker, was undetectable (Fig. 1B); GAPDH has been reported to localize in mitochondria [31]. Our study showed a rapid mitochondrial p53 increase at 1 h that remained throughout the 24-h time course post TPA treatment (Fig. 1A, lower panel). Consistent with the p53 mitochondrial translocation, correspondent changes in mitochondrial functions were also observed, which included increased ROS generation, loss of mitochondrial complex I activity, decreased mitochondrial membrane potential, and release of intermembrane cytochrome *c* (Fig. 1C∼1F). Moreover, the activation of p53 was associated with increases in the expression levels of Bax, a pro-apoptotic Bcl-2 protein which can trigger the MOM permeabilization in cancer cell apoptosis [32], as well as, increases in DNA fragmentation (Fig. 1G&1H).

To further confirm the correlation between p53 activation and downstream responses including mitochondrial dysfunction and apoptosis, a designed p53-targeted shRNA sequence-containing plasmid was introduced into JB6 P+ cells. In transfected cells, p53 expression was significantly inhibited, and TPA-induced p53 accumulation and mitochondrial translocation were suppressed (Fig. 1I). As expected, p53 knock down cells did not respond to TPA treatment in both mitochondrial functions (Fig. 1C–1F) and apoptotic cell death (Fig. 1G&1H). These results showed that presence of p53 is critical for TPA-induced mitochondrial dysfunction and apoptosis in JB6 P+ cells.

### Mitochondrial uncoupling suppressed TPA-induced p53 mitochondrial translocation in JB6 P+ cells

**Figure 2:**
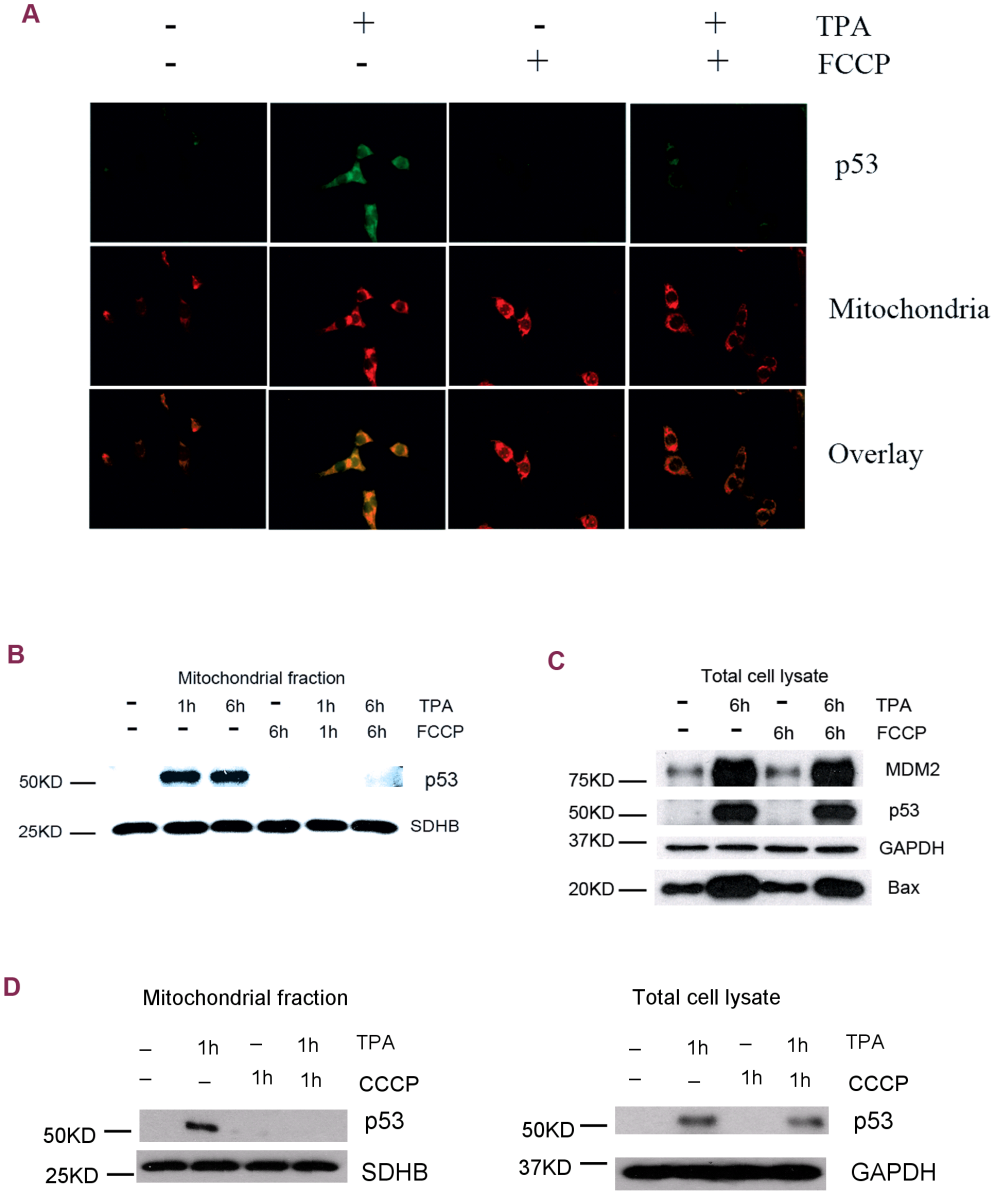
Mitochondrial uncoupling suppressed TPA-induced p53 mitochondrial translocation in JB6 P+ cells. (A) Immunofluorescent staining of p53 at 30 min after TPA treatment. First panel: p53 detected with an anti-p53 antibody. Second panel: mitochondria labeled with MitoTracker Red. Third panel: overlay pictures. (B) p53 mitochondrial translocation. SDHB served as the loading control. (C) Cellular p53 activation and downstream gene expression. GAPDH served as the loading control. FCCP: 10 µM. (D) Western blot analysis of the mitochondrial (left) and the whole cellular (right) p53 levels after CCCP treatment. CCCP: 5 µM.

The above studies show that the tumor promoter TPA induces p53 mitochondrial translocation and subsequent mitochondrial dysfunction. Next, we examined whether mitochondrial uncoupling, in turn, affected p53 mitochondrial translocation and subsequent apoptosis. Indeed, immunofluorescent staining studies demonstrated that TPA-induced p53 mitochondrial translocation was extensively blocked by the protonophore FCCP, which is known to be able to uncouple oxidative phosphorylation by carrying protons across the mitochondrial membrane. As shown in Figure 2A, 30 min after TPA treatment, a fraction of p53 (green signal) was detected in the mitochondria which colocalized with the mitochondrial marker-MitoTracker Red. This mitochondrial accumulation of p53 was significantly reduced by FCCP co-treatment. These results were further confirmed using Western blot analysis. The induced accumulation of p53 in mitochondria was fully blocked by FCCP co-treatment at both 1 h and 6 h after TPA treatment (Fig. 2B). Interestingly, in contrast to the effect of FCCP in the mitochondria, there was no significant reduction in TPA-induced cellular p53 accumulation and transcriptional activation of MDM2 and Bax by FCCP co-treatment (Fig. 2C). Taken together, the effect of mitochondrial uncoupling seems to be specific for mitochondrial p53 accumulation and non-transcriptional p53 activities. Another mitochondrial uncoupling reagent, CCCP, also showed results similar to FCCP treatment. CCCP co-treatment suppressed TPA-induced p53 mitochondrial translocation with no dramatic effects on the whole cellular p53 levels (Fig. 2D).

### Mitochondrial uncoupling suppressed TPA-induced p53 mitochondrial translocation in 308 cells

**Figure 3:**
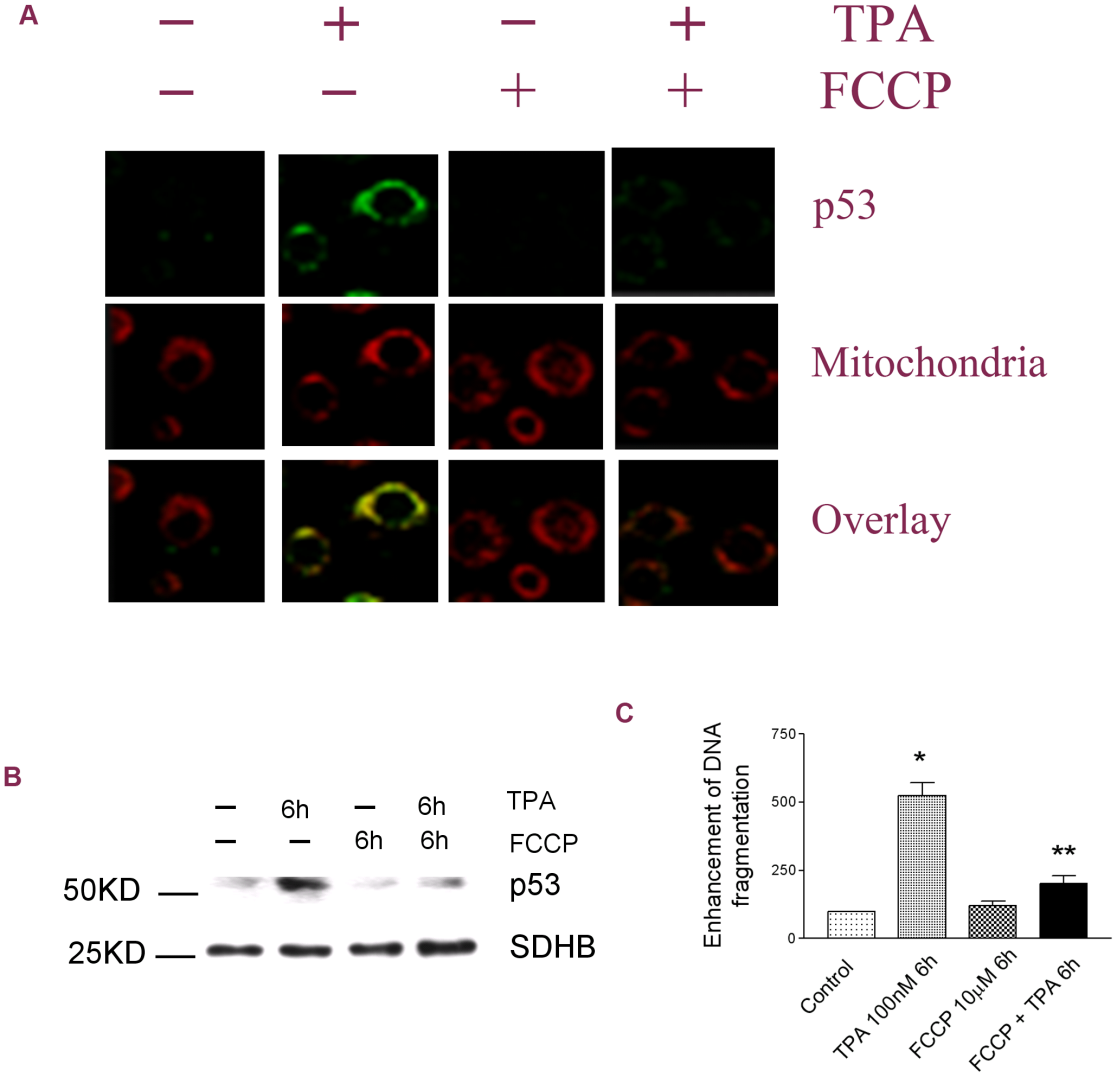
Mitochondrial uncoupling suppressed TPA-induced p53 mitochondrial translocation in 308 cells. (A) Immunofluorescent staining of p53 at 30 min after TPA treatment. First panel: p53 detected with an anti-p53 antibody. Second panel: mitochondria labeled with MitoTracker Red. Third panel: overlay pictures. (B) p53 mitochondrial translocation. SDHB served as the loading control. (C) Apoptotic cell death revealed by the DNA fragmentation ELISA assay. *: Significant difference compared to the control. **: Significant difference compared to TPA treatment.

Our previous studies have demonstrated that TPA can induce p53 activation and mitochondrial translocation in 308 cells [23]. Here we examined whether mitochondrial uncoupling also affects p53 mitochondrial translocation and subsequent apoptosis in this cell model. As shown in Fig. 3A, similar to the results in JB6 cells, TPA treatment induced rapid (30 min after TPA treatment) mitochondrial translocation of p53 as indicated by immunofluorescent staining studies, and this p53 translocation was blocked by FCCP co-treatment. These results were further confirmed using Western blot analysis. The mitochondrial p53 levels were suppressed by FCCP co-treatment 6 h after TPA treatment (Fig. 3B). Not surprisingly, TPA-induced apoptotic cell death was also suppressed by FCCP cotreatment (Fig. 3C).

### Mitochondrial uncoupling prevented mitochondrial dysfunction and apoptosis associated with p53 mitochondrial translocation in JB6 P+ cells

**Figure 4:**
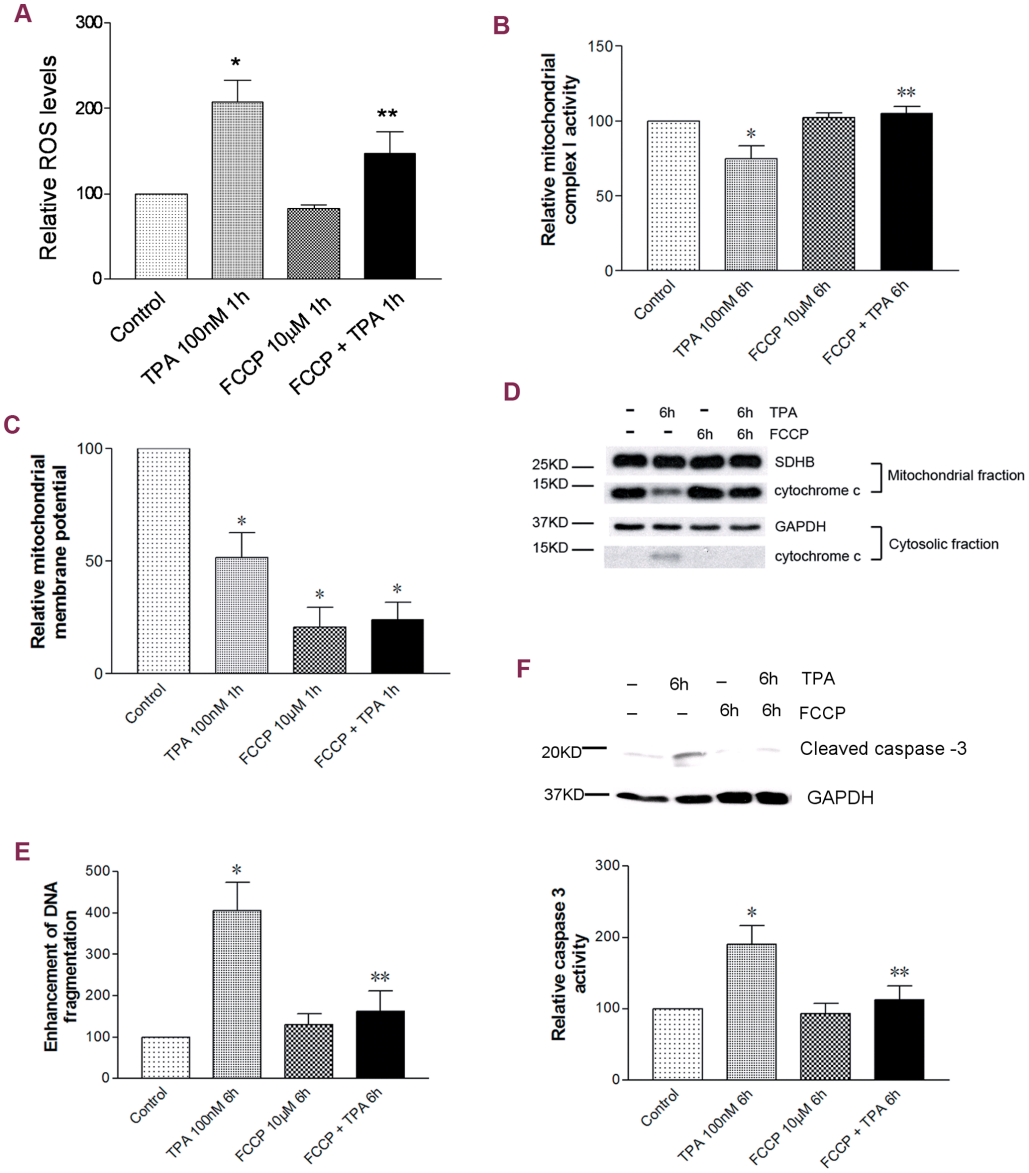
Mitochondrial uncoupling prevented mitochondrial dysfunction and apoptosis associated with p53 mitochondrial translocation in JB6 P+ cells. (A) ROS levels. (B) Mitochondrial complex I activities. (C) Mitochondrial membrane potential. (D) Cytochrome *c* detected in both mitochondrial and cytosolic fractions. GAPDH or SDHB served as the loading control. (E) Cell death levels revealed by the DNA fragmentation ELISA assay. (F) Apoptosis revealed by detection of cleave caspase 3 and caspase 3 activity assay. *: Significant difference compared to the control. **: Significant difference compared to TPA treatment.

Given the strong correlation between p53 and downstream apoptotic events, we further examined whether blocking p53 mitochondrial translocation by mitochondrial uncoupling affected mitochondrial functions and apoptosis. As shown in Figure 4, FCCP alone caused a strong decrease in mitochondrial membrane potential; whereas, no obvious effects on the ROS level, Complex I activity and cytochrome *c* levels were observed. However, FCCP co-treatment significantly attenuated or blocked TPA-induced rapid ROS generation (Fig. 4A), the loss of Complex I activity (Fig. 4B), and mitochondrial cytochrome *c* release (Fig. 4D). Although the effect of FCCP on TPA-induced decrease in mitochondrial membrane potential was unable to be detected because of the extensive dissipating effect of FCCP alone (Fig. 4C), our current data still indicates that mitochondrial uncoupling exerts an efficient inhibiting effect on p53-associated mitochondrial dysfunction.

Consistent with the conservation of mitochondrial function and integrity by mitochondrial uncoupling, TPA-induced apoptosis was also attenuated by FCCP co-treatment as detected using the Cell Death ELISA and cleaved caspase 3 activity assay (Fig. 4E, 4F).

### UCP2-knockdown JB6 P+ cells showed enhanced p53 mitochondrial translocation and decreased anchorage-independent growth in response to TPA treatment

**Figure 5:**
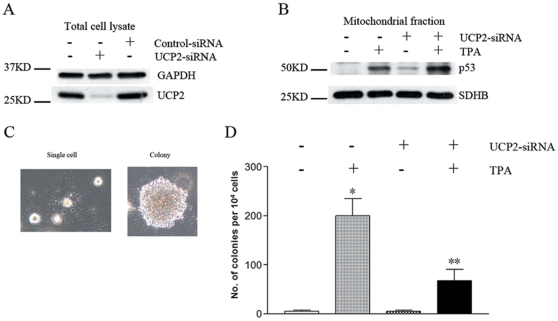
UCP2 knockdown enhanced p53 mitochondrial translocation and decreased colony formation in response to TPA treatment. (A) UCP2 knockdown efficiency examined by Western blot analysis. (B) p53 mitochondrial translocation. (C) Soft agar colony formation assays of JB6 P+ cells (light micrograph: 100× magnification). (D) Quantification of formed colonies. Cells grown in 0.33% soft agar containing 6.66 ng/ml TPA or vehicle (DMSO 1000× diluted). *: Significant difference compared to DMSO treatment. **: Significant difference compared to normal cells with TPA treatment.

It is notable that a mild uncoupling effect is present in most mammal cells. Ample evidence has indicated that this mild uncoupling has multiple physiological roles including export of fatty acid anions from mitochondria, regulation of insulin secretion, as well as, attenuation of mitochondrial superoxide production [33]. There are a series of specific uncoupling proteins (UCP1–5) in mammal cells. Among which, UCP2 is widely distributed in various tissues and critical for ROS regulation, as well as, survival of tumor cells [19], [33]. Given these facts, we further examined the role of UCP2 in TPA-induced p53 mitochondrial translocation and cell transformation of JB6 P+ cells. UCP2 gene expression was knocked down using a specific siRNA transfection approach (Fig. 5A). As shown in Fig. 5B, UCP2 absence induced a small amount of p53 mitochondrial translocation, and also enhanced TPA-induced p53 mitochondrial translocation. Moreover, the soft agar (cell transformation) assay demonstrated a highly dependence on the presence of UCP2, since the UCP2-knockdown cells were much less prone to form colonies compared to the control JB6 P+ cells (Fig. 5C,5D). These data indicated that UCP2-mediated mitochondrial uncoupling may contribute to TPA-induced tumor transformation of JB6 P+ cells.

## Discussion

Our previous studies have demonstrated that during early skin carcinogenesis, increased apoptotic cell death is associated with increased cell proliferation [20]. As an important apoptotic signal, the tumor suppressor p53 was activated as indicated by increased expression levels, DNA binding activity, expression of its target genes, and moreover, translocation to mitochondria [20], [22]. Potentially, as a defending mechanism against carcinogenesis, p53 induces apoptosis via transcription-dependent and transcription-independent pathways, two fundamentally different, but synergistic mechanisms of action [16], [34], [35]. In the nucleus, p53 regulates the transcription of proapoptotic effectors such as Bax, Puma and Noxa. Meanwhile, a fraction of p53 translocates to mitochondria where it directly interacts with mitochondrial proapoptotic proteins such as p53AIPI [36] and multi-domain members of the Bcl2 family [37], as well as, the antioxidant defense enzyme, manganese superoxide dismutase [22].

Mitochondria are the power house of the cell, and ATP is generated through the mitochondrial electron transport chain. How p53 affects major mitochondrial respiration has not been well studied. On the other hand, changes in mitochondrial functions will likely modulate p53 mitochondrial translocation as well. Our previous studies have suggested that the mitochondrial permeability transition pore (PTP) could serve as both the cause and barrier for p53 mitochondrial translocation, since mitochondrial accumulation of p53 induces PTP opening; whereas blocking the PTP by cyclosporine A suppresses p53 translocation to mitochondria [38]. Our current study aims to investigate if the mitochondrial electron transport chain is uncoupled during early cancer development, and whether mitochondrial uncoupling affects p53 translocation to mitochondria. Our results suggest that chemically uncoupling oxidative phosphorylation in mitochondria is able to block p53 mitochondrial translocation; and, in turn, antagonize most of the apoptotic downstream events including rapid ROS generation, mitochondrial cytochrome *c* release, loss of mitochondrial Complex I activity, and finally apoptosis.

Our results may also help understand the effects of mitochondrial uncoupling in anti-cancer therapy. It is well known that cancer cells have acquired a metabolic reprogramming to fermentation which can bypass the Pasteur Effect (the Warburg Effect) [39]. Recently, this kind of “respiratory defect” is associated with mitochondrial uncoupling [7] based on the facts that UCP2, one of the major uncoupling proteins in mammals, is overexpressed in various chemoresistant cancer cell lines and primary human cancer samples; and that overexpression of UCP2 leads to an increased apoptotic threshold [19]. Moreover, mitochondrial uncoupling is reported to be able to oppose the onset of apoptosis in several human cancer cell lines [18], [19], [40]. Our results suggest that the contribution of UCP2 in cancer cells might be more complicated than described before. In our studies, the UCP2 knockdown cells show a stronger response to TPA-induced p53 mitochondrial translocation than the control cells by being less prone to form colonies in response to TPA treatment in the soft agar assay. These results suggest that UCP2 may play an important role in both transformed and transforming cells. The overexpression of UCP2 in cancer cells may be a result of a long-term selecting procedure during transformation, since any mutant that results in UCP2 upregulation could help cells escape from apoptosis mastered via the p53 network. Given the fact that mitochondrial uncoupling could cause dissipation of the mitochondrial potential, a decrease of mitochondrial ROS generation, and the sensitivity of p53 activation to the intrinsic redox balance [33], [19], [24], it is reasonable to hypothesize that mitochondrial uncoupling may provide malignant cells with a prosurvival advantage by interfering with the p53-mediated apoptosis pathway.

The present investigation suggests yet another relationship between mitochondrial uncoupling, p53 mitochondrial translocation, and p53-induced apoptosis during early skin tumor promotion in JB6 P+ and 308 cells. Our data demonstrated that mitochondrial uncoupling efficiently prevented TPA-induced mitochondrial ROS generation, and blocked p53 mitochondrial translocation, leading to prevention of mitochondrial dysfunction and attenuation of apoptotic cell death. Taken together, our studies suggest a novel mechanism to explain why mitochondrial uncoupling, as a major physiologic phenomenon during carcinogenesis, may contribute to the viability and chemoresistance of tumor cells; which is mediated at least in part, by converting a mitochondrial physiological condition into a unique status. Thus, mitochondrial uncoupling maintains ROS at a reasonable level and interrupts the p53-ROS positive feed-back loop which further prevents p53 mitochondrial translocation, associated mitochondrial dysfunction, and p53-mediated apoptosis.

In summary, our findings indicate that mitochondrial uncoupling modulates p53 mitochondrial translocation and associated apoptosis pathways during skin tumor promotion. Therefore, targeting mitochondrial uncoupling may be considered as a novel treatment strategy for cancer.
